# Ablation of Galectin-12 Inhibits Atherosclerosis through Enhancement of M2 Macrophage Polarization

**DOI:** 10.3390/ijms21155511

**Published:** 2020-07-31

**Authors:** En-Shyh Lin, Yu-An Hsu, Ching-Yao Chang, Hui-Ju Lin, Chih Sheng Chen, Lei Wan

**Affiliations:** 1 Department of Beauty Science, National Taichung University of Science and Technology, Taichung 403, Taiwan; eslin@nutc.edu.tw; 2 School of Chinese Medicine, China Medical University, Taichung 404, Taiwan; annhsu007@gmail.com (Y.-A.H.); irisluu2396@gmail.com (H.-J.L.); 3 Department of Biotechnology, Asia University, Taichung 413, Taiwan; cychang@asia.edu.tw; 4 Department of Ophthalmology, China Medical University Hospital, Taichung 404, Taiwan; 5 Division of Chinese Medicine, Asia University Hospital, Taichung 413, Taiwan; 6 Department of Food Nutrition and Health Biotechnology, Asia University, Taichung 413, Taiwan; 7 Department of Chinese Medicine, China Medicine University Hospital, Taichung 404, Taiwan; 8 Department of Obstetrics and Gynecology, China Medical University Hospital, Taichung 404, Taiwan

**Keywords:** atherosclerosis, foam cell, galectin-12, low-density lipoprotein, macrophage polarization

## Abstract

The formation of foam cells, which are macrophages that have engulfed oxidized low-density lipoprotein (OxLDL), constitutes the first stage in the development of atherosclerosis. Previously, we found that knocking down galectin-12, a negative regulator of lipolysis, leads to reduced secretion of monocyte chemoattractant protein-1 (MCP-1), a chemokine that plays an important role in atherosclerosis. This prompted us to study the role of galectin-12 in atherosclerosis. With that aim, we examined foam cell formation in Gal12^‒/‒^ murine macrophages exposed to OxLDL and acetylated LDL (AcLDL). Then, we generated an LDL receptor and galectin-12 double knockout (DKO) mice and studied the effect of galectin-12 on macrophage function and atherosclerosis. Lastly, we evaluated the role of galectin-12 in human THP-1 macrophages using a doxycycline-inducible conditional knockdown system. Galectin-12 knockout significantly inhibited foam cell formation in murine macrophages through the downregulation of cluster of differentiation 36 (*CD36*), and the upregulation of ATP Binding Cassette Subfamily A Member 1 (*ABCA1*), ATP Binding Cassette Subfamily G Member 1 (*ABCG1*), and scavenger receptor class B type 1 (*SRB1*). Consistent with this, galectin-12 knockdown inhibited foam cell formation in human macrophages. In addition, the ablation of galectin-12 promoted M2 macrophage polarization in human and murine macrophages as evidenced by the upregulation of the M2 marker genes, *CD206* and *CD163,* and downregulation of the M1 cytokines, tumor necrosis factor α (TNF- α), interleukin-6 (IL-6), and MCP-1. Moreover, the ablation of galectin-12 decreased atherosclerosis formation in DKO mice. Based on these results, we propose galectin-12 as a potential therapeutic target for atherosclerosis.

## 1. Introduction

Atherosclerosis is considered a chronic disease involving a persistent inflammatory response to injured arterial walls, which may be caused by dyslipidemia, diabetes, and/or hypertension [1,2]. In the process of atherosclerosis, endothelial dysfunction results in the accumulation of fat in arterial walls, activating innate and adaptive immune responses that lead to inflammation [3]. The increased permeability of the dysfunctional endothelium facilitates low-density lipoprotein (LDL) migration into the subendothelial space. Then, the infiltrated LDLs are oxidized and subsequently engulfed by cells expressing scavenger receptors, such as macrophages. Fatty streaks, the earliest detectable atherosclerotic lesions, are predominantly composed of macrophage foam cells [3,4,5,6], which are macrophages that have taken up large amounts of OxLDL. The stimulation of infiltrated monocytes in the subendothelial region by OxLDL-induced superoxides results in the polarization of macrophages towards a proinflammatory (M1) phenotype. M1 macrophages secrete TNF-α and IL-6, which cause endothelial dysfunction. Thus, the vicious cycle formed between OxLDL and the inflammatory cytokines (TNF-α and IL-6) from macrophages in the subendothelial space results in a persistent inflammatory response that promotes atherosclerosis [4]. In a previous study, we found that the ablation of galectin-12 decreased the secretion of MCP-1, TNF-α, and IL-6 in lipopolysaccharide (LPS)—and saturated fatty-acid-stimulated macrophages [7,8], suggesting that galectin-12 may potentially play a role in the pathogenesis of atherosclerosis.

Galectin-12 is predominantly expressed in adipose tissue and also detected in low amounts in the heart, pancreas, spleen, thymus, and peripheral blood leukocytes [9,10,11,12,13]. Galectin-12 is gaining increased attention because of its involvement in the regulation of lipid metabolism, insulin sensitivity, and glucose tolerance. Insulin sensitivity is associated with chronic inflammation and the complex regulation of adipocytes and macrophages. Recently, it was found that galectin-12 is predominantly located in large lipid droplets, and that it functions as a negative regulator of lipolysis. Lipolysis is enhanced in galectin-12 knockout mice through increased protein kinase A (PKA) activity and cyclic adenosine monophosphate (cAMP) levels. Moreover, galectin-12 deficiency improves insulin sensitivity and glucose tolerance in overweight animals [14]. The ablation of galectin-12 also results in polarization of macrophages into the M2 subset. The negative regulation of M2 macrophage polarization by galectin-12 causes enhanced inflammation and decreases insulin sensitivity in adipocytes [8]. However, it remains unknown whether galectin-12 knockout could ameliorate atherosclerosis through the downregulation of chronic inflammation. Thus, we sought to clarify the mechanisms whereby galectin-12 participates in the pathogenesis of atherosclerosis.

## 2. Results

**Figure 1:**
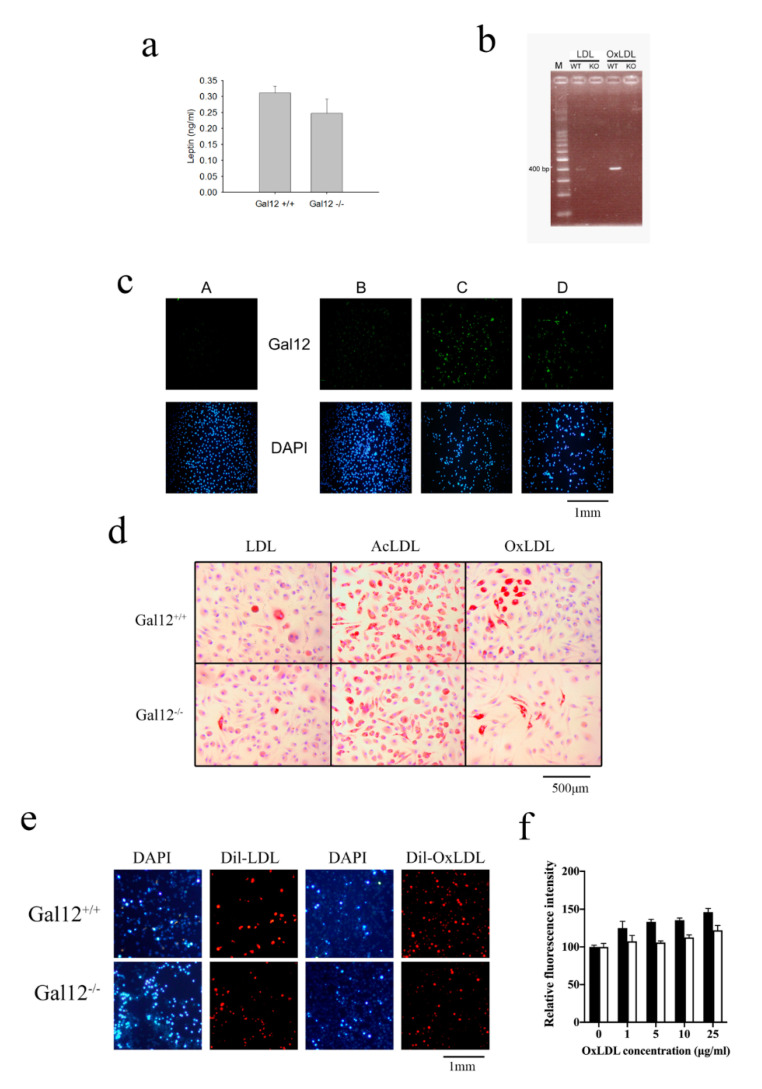
Oxidized low-density lipoprotein (OxLDL) and acetylated LDL (AcLDL) altered galectin-12 expression level and foam cell formation in bone-marrow-derived macrophages. (**a**) Ablation of galectin-12 lowered the expression level of leptin in Gal12^‒/‒^ mice sera. Sera were collected from 10-week old Gal12^+/+^ and Gal12^‒/‒^ mice and leptin concentrations were determined using ELISA kits; (**b**) OxLDL treatment increased the expression level of galectin-12 in WT (Gal12^+/+^) bone-marrow-derived macrophages (BMDM). WT and KO (Gal12^‒/‒^) BMDM were treated with 100 μg/mL OxLDL for 4 h, and then the expression level of galectin-12 was determined; (**c**) AcLDL and OxLDL treatment increased the expression level of galectin-12 in Gal12^+/+^ BMDM. Galectin-12 was identified using immunofluorescence staining, and cell nuclei were stained with 4′,6-diamidino-2-phenylindole (DAPI). A: LDL-treated Gal12^+/+^ BMDM stained with antimouse IgG Alexa Fluor 488-labeled secondary antibody as control; B-D: Gal12^+/+^ BMDM treated for 24 h with 100 μg/mL LDL (B); 100 μg/mL AcLDL (C); 100 μg/mL OxLDL (D); (**d**) Gal12^+/+^ and Gal12^‒/‒^ BMDM were treated with 100 μg/mL LDL, 100 μg/mL AcLDL or 100 μg/mL OxLDL for 24 h and foam cell formation was detected using Oil Red O staining; (**e**) Gal12^+/+^ and Gal12^‒/‒^ BMDM were treated with 20 μg/mL dil-LDL and dil-OxLDL to determine foam cell formation. Cell nuclei were stained with DAPI. Lipid droplet-containing macrophages were detected using fluorescent microscopy; (**f**) Gal12^+/+^ (black column) and Gal12^‒/‒^ (white column) BMDM were seeded in 96-well plates and different concentrations of OxLDL were added to promote foam cell formation. Foam cell formation was determined using AdipoRed^TM^ staining and a fluorometer for detection.

**Figure 2:**
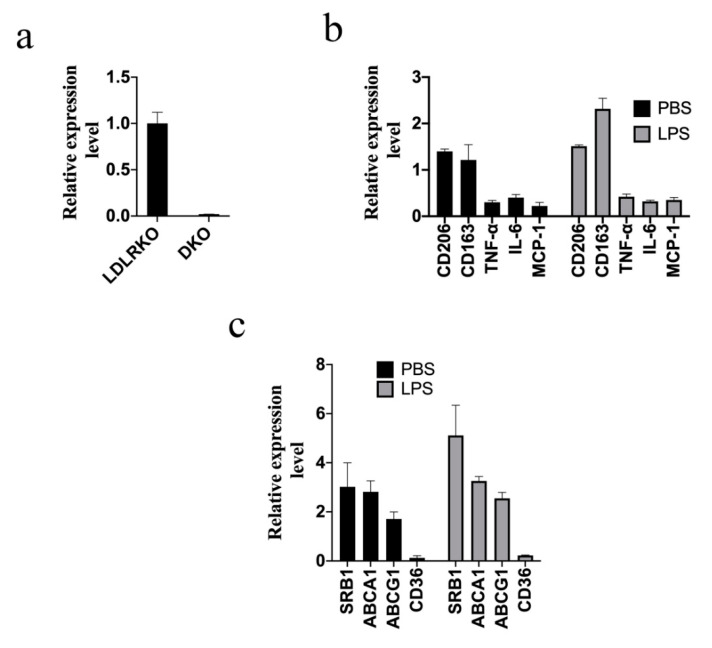

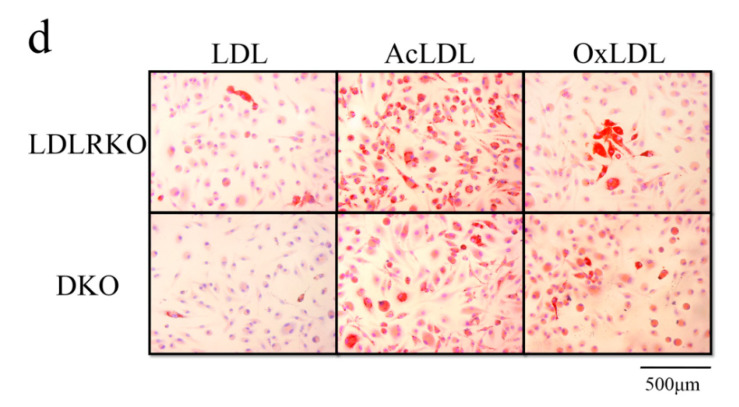
Ablation of galectin-12 in LDL receptor knockout (LDLRKO) mice enhanced M2 macrophage polarization and inhibited foam cell formation. (**a**) Double knockout (DKO) BMDM did not express galectin-12 as determined using quantitative RT-PCR. The expression level of galectin-12 in LDLRKO (expression set as 1) was used to normalize the expression level in DKO mice; (**b**) DKO BMDM tended toward M2 macrophage polarization. LDLRKO and DKO BMDM were treated with PBS or 100 ng/mL lipopolysaccharide (LPS) for 24 h and the expression levels of *CD163*, *CD206*, *TNF-α*, *IL-6*, and *MCP-1* were determined using quantitative RT-PCR. The expression levels of *CD163*, *CD206*, *TNF-α*, *IL-6* and *MCP-1* in LDLRKO (expression set as 1, not shown) were used to normalize the levels in DKO mice; (**c**) DKO BMDM expressed higher levels of cholesterol efflux genes (*SRB1*, *ABCA1*, and *ABCG1*), and lower levels of the cholesterol influx gene *CD36*. Levels were quantified using quantitative RT-PCR. The expression levels of *SRB1*, *ABCA1*, *ABCG1*, and *CD36* in LDLRKO (expression set as 1, not shown) were used to normalize the expression levels in DKO mice; (**d**) LDLRKO and DKO BMDM were treated with 100 μg/mL LDL, 100 μg/mL AcLDL, or 100 μg/mL OxLDL for 24 h and foam cell formation was detected using Oil Red O staining.

**Figure 3:**
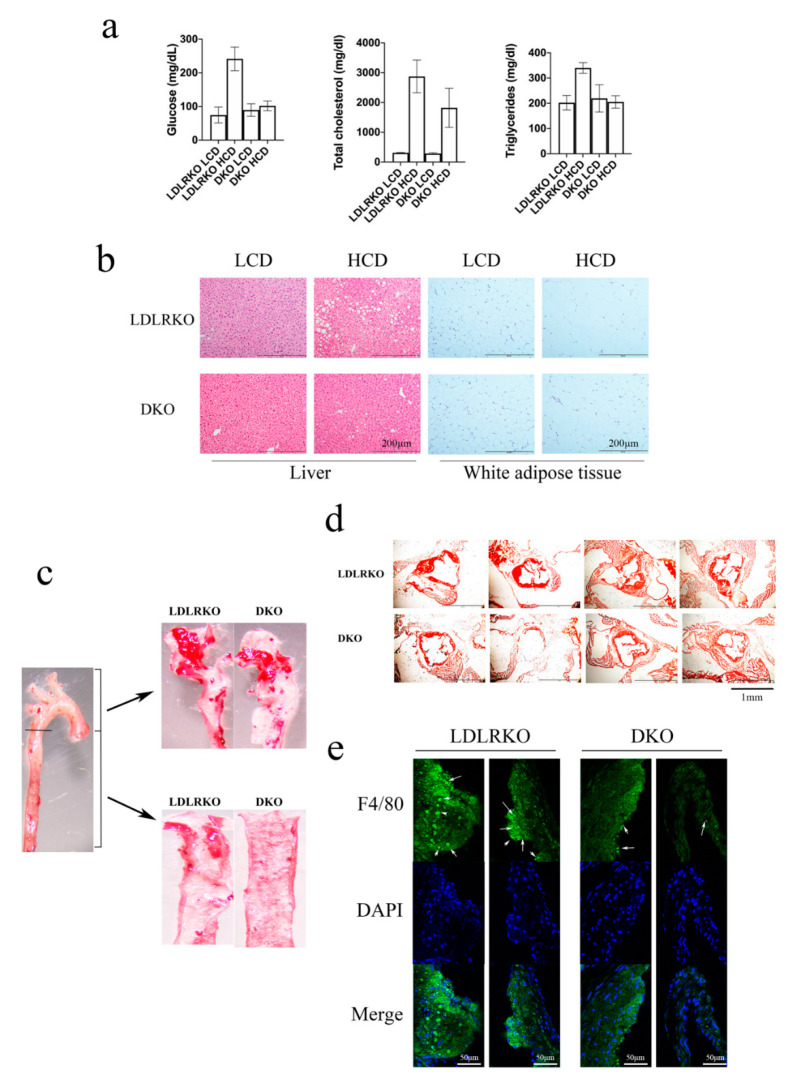
Galectin-12 deficiency reduces adiposity, fatty liver, and atherosclerotic lesions. Mice were fed with a western diet for 12 weeks, and tissues were harvested for the following experiments. (**a**) High-cholesterol diet (HCD)-fed DKO mice had significantly lower blood glucose, serum total cholesterol, and triglycerides than HCD-fed LDLRKO mice; (**b**) H&E staining of paraffin sections of epididymal fat depots and liver tissues from LDLRKO and DKO mice (representative of four experiments); (**c**) Oil Red O staining of aortic arch and descending thoracic aorta from HCD-fed LDLRKO and DKO mice (representative of four experiments); (**d**) Oil Red O staining of aortic roots from LDLRKO and DKO mice. The sections presented were from different mice; (**e**) Macrophage infiltration (white arrow) into the aortic roots of HCD-fed LDLRKO and DKO mice was revealed using anti-F4/80 staining. Cell nuclei were stained with DAPI. Macrophages in the aortic roots were identified using fluorescent microscopy.

To determine whether galectin-12 may be involved in the pathogenesis of atherosclerosis, we measured leptin concentration in the sera of galectin-12 wild type (Gal12^+/+^) and knockout (Gal12^−/−^) mice. The expression level of leptin, a cytokine that has a known role in the pathogenesis of atherosclerosis, was significantly lower in Gal12^−/−^ mice than in Gal12^+/+^ (Figure 1a). We further found that treatment with OxLDL or acetylated low-density lipoprotein (AcLDL) increased the level of galectin-12 in Gal12^+/+^ bone-marrow-derived macrophages (BMDM; Figure 1b,c). We further tested foam cell formation ability in Gal12^+/+^ and Gal12^−/−^ BMDM using three different approaches (1) Oil Red O staining (Figure 1d), (2) dil-OxLDL staining (Figure 1e), and (3) AdipoRed^TM^ staining (Figure 1f). There were no significant differences in foam cell formation between Gal12^+/+^ and Gal12^−/−^ BMDM in LDL-treated cells (Figure 1d,e). However, upon treatment with 100 μg/mL AcLDL or 100 μg/mL OxLDL, a significantly higher proportion of lipid droplets was found in Gal12^+/+^ versus Gal12^−/−^ BMDM (2.8 ± 0.8, and 3.4 ± 0.5-fold higher upon AcLDL and OxLDL treatment, respectively; Figure 1d). Consistent with the previous results, we found a 3.2 ± 0.4-fold higher foam cell formation in dil-OxLDL stained Gal12^+/+^ versus Gal12^−/−^ BMDM (Figure 1e). Moreover, foam cells stained with AdipoRed^TM^ revealed higher lipid content in Gal12^+/+^ versus Gal12^−/−^ BMDM (Figure 1f). Together, the results revealed lower foam cell formation in Gal12^−/−^ BMDM indicating that the inhibition of galectin-12 may be beneficial in the treatment of atherosclerosis.

To understand the role of galectin-12 in the pathogenesis of atherosclerosis, LDL receptor knockout (LDLRKO) and galectin-12 double knockout (DKO) mice were generated. As shown in Figure 2a, no detectable expression of galectin-12 (Figure 2a) was found in BMDM isolated from DKO mice. Next, we used quantitative RT-PCR to examine BMDM polarization in the presence and absence of LPS stimulation. As shown in Figure 2b, we found higher levels of M2 markers (*CD206* and *CD163*) and lower levels of M1 markers (*TNF-α*, *IL-6*, and *MCP-1*) in DKO BMDM than in LDLRKO BMDM, indicating M2 macrophage polarization in DKO macrophages. Then, to understand the mechanisms involved in lipid accumulation, the expression levels of genes involved in cholesterol influx/efflux were determined in BMDM in the presence and absence of LPS stimulus. We found lower levels of *CD36* and significantly higher levels of *SRB1*, *ABCA1*, and *ABCG1* in DKO BMDM compared with LDLRKO BMDM (Figure 2c). The results suggested that galectin-12 knockout may reduce lipid accumulation through the inhibition of *CD36*, which is involved in cholesterol influx in macrophages, and the promotion of *SRB1*, *ABCA1*, and *ABCG1*, which enhance cholesterol efflux (Figure 2d). Treatment with AcLDL or OxLDL resulted in 4.1 ± 0.9-fold and 2.2 ± 0.4-fold higher foam cell formation in LDLRKO BMDM versus DKO, respectively (Figure 2d), indicating that the ablation of galectin-12 suppresses the foam cell formation induced by AcLDL and OxLDL.

Next, LDLRKO and DKO mice were fed with either a low-cholesterol diet (LCD) or a high-cholesterol diet (HCD) for 12 weeks to induce atherosclerosis. We found no difference either in the amount of food ingested between LCD and HCD-fed mice, or in weight gain between LDLRKO and DKO mice (data not shown). We found a significantly lower fasting blood glucose in HCD-fed DKO mice compared with HCD-fed LDLRKO mice (Figure 3a). In HCD-fed DKO mice, total cholesterol and triglycerides were also significantly lower than in HCD-fed LDLRKO mice (Figure 3a). The liver of HCD-fed LDLRKO mice showed immense lipid droplets, a condition that was abolished with the knockout of galectin-12 (Figure 3b). Moreover, the white adipocytes of HCD-fed DKO mice were much smaller than those of HCD-fed LDLRKO mice (Figure 3b). In addition, the ablation of galectin-12 induced a significant decrease in atherosclerotic lesion size in the aortic arch and the descending thoracic aorta of mice fed HCD (Figure 3c). Specifically, the ablation of galectin-12 significantly reduced atheroma formation in the aortic arch (2.48-fold reduction) and the descending thoracic aorta (8.14-fold reduction) in DKO mice compared with LDLRKO mice (Figure 3c). Moreover, we found a 53.2% reduction in lesion size at the aortic root in HCD-fed DKO mice compared to HCD-fed LDLRKO mice (Figure 3d). In addition, the aortic root of HCD-fed DKO mice displayed significantly lower macrophage infiltration (3.38 ± 0.46-fold decrease) compared to the aortic root of HCD-fed LDLRKO mice (Figure 3e), indicating that galectin-12 deletion significantly reduced macrophage infiltration.

**Figure 4:**
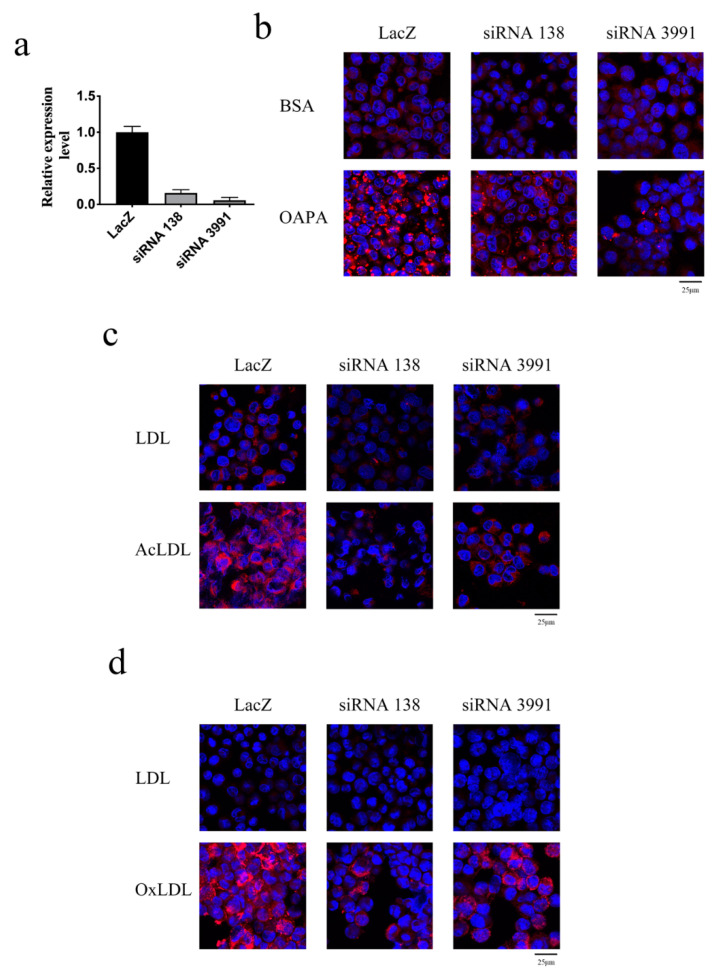
Galectin-12 knockdown inhibited foam cell formation in human macrophages. (**a**) THP-1 cells from different groups were treated with doxycycline for 72 h and the galectin-12 expression levels were determined using quantitative RT-PCR; (**b**–**d**) THP-1 cells were treated with 1 μg/mL doxycycline for 24 h and then with 100 ng/mL phorbol 12-myristate 13-acetate + 1 μg/mL doxycycline for another 48 h to promote macrophage differentiation. Differentiated THP-1 macrophages were treated with 100 μg/mL oleic acid + 100 μg/mL palmitic acid (OAPA) (**b**), 100 μg/mL AcLDL (**c**), or 100 μg/mL OxLDL (**d**) for 24 h and then foam cell formation was analyzed using AdipoRed^TM^ staining.

Next, to evaluate the role of galectin-12 in human macrophages, we applied a doxycycline-inducible conditional knockdown system in the human THP-1 cell line. LacZ gene knockdown (control) and two different galectin-12 gene knockdown cells were generated. The expression levels of galectin-12 in siRNA 138 and siRNA 3991 knockdown cells were significantly decreased when treated with 1 μg/mL doxycycline for 72 h (Figure 4a). In macrophages treated with a combination of oleic acid and palmitic acid (OAPA), there were 2.78 ± 0.25-fold and 6.27 ± 0.38-fold lower lipid droplet formation in siRNA 138 and siRNA 3991 galectin-12 knockdown macrophages compared to control, respectively (Figure 4b). In addition, galectin-12 knockdown macrophages exhibited reduced lipid droplets compared to control cells when treated with either AcLDL (Figure 4c; 8.27 ± 0.39-fold and 3.98 ± 0.78-fold lower in siRNA 138 and siRNA3991 macrophages, respectively) or OxLDL (Figure 4d; 5.48 ± 0.33-fold and 2.97 ± 0.49-fold lower in siRNA 138 and siRNA3991 macrophages, respectively).

**Figure 5:**
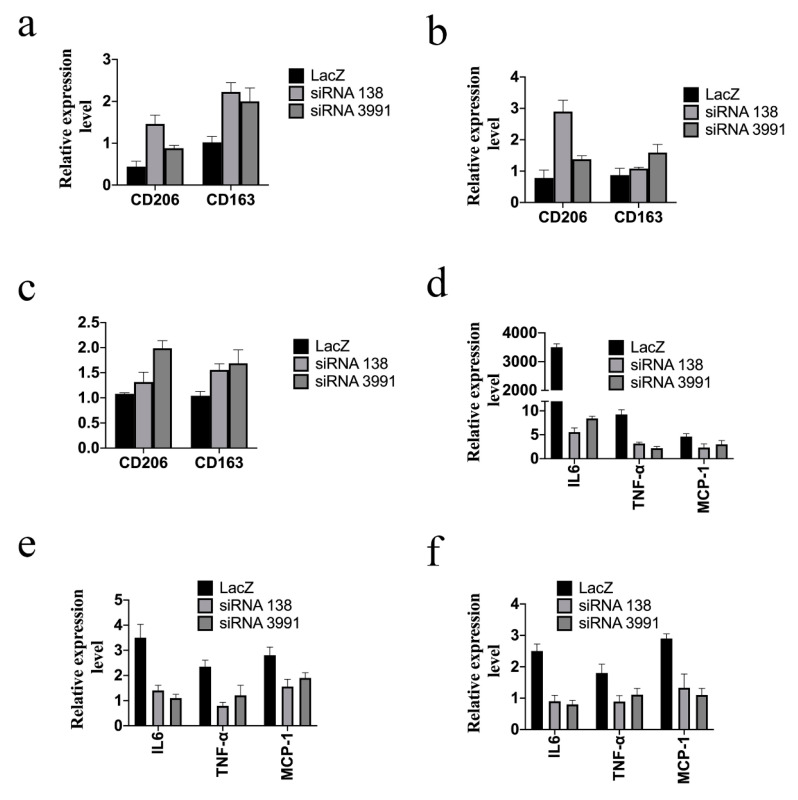

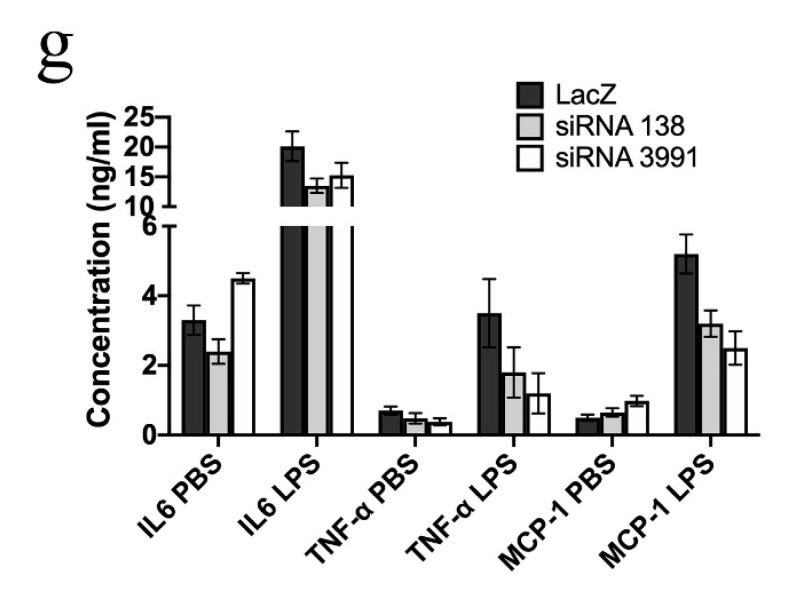
Galectin-12 knockdown enhances M2 macrophage polarization of human macrophages. (**a**–**c**) Galectin-12 knockdown differentiated THP-1 macrophages expressed lower *CD206* and *CD163* RNA levels when activated with 100 ng/mL LPS (**a**), 100 μg/mL OAPA (**b**), or 100 μg/mL OxLDL (**c**). The expression levels of *CD206* and *CD163* in PBS-treated THP-1 macrophages (expression set as 1; not shown) were used to normalize the expression levels in LPS-, OAPA-, and OxLDL-treated macrophages. RNA was quantified using quantitative RT-PCR; (**d**–**f**) Galectin-12 knockdown differentiated THP-1 macrophages expressed lower *IL-6*, *TNF-α* and *MCP-1* RNA levels when activated with 100 ng/mL LPS (**d**), 100 μg/mL OAPA (**e**), or 100 μg/mL OxLDL (**f**). The RNA expression levels of *IL-6*, *TNF-α* and *MCP-1* in PBS-treated THP-1 macrophages (expression set as 1; not shown) were used to normalized the RNA expression levels in LPS-, OAPA- and OxLDL-treated macrophages. RNA was quantified using quantitative RT-PCR; (**g**) Galectin-12 knockdown differentiated THP-1 macrophages expressed lower IL-6, TNF-α, and MCP-1 cytokine levels when activated with LPS. Cytokine levels were measured using commercial ELISA kits.

The mRNA levels of M2 markers (*CD163* and *CD206*) among LacZ, siRNA 138, and siRNA 3991 macrophages were also determined. Galectin-12 knockdown THP-1 macrophages activated with LPS (Figure 5a), OAPA (Figure 5b) or OxLDL (Figure 5c) showed higher expression of M2 markers than the LacZ cells. The results indicated that the knockdown of galectin-12 in human macrophages promoted M2 macrophage polarization. The gene expression levels of *IL-6*, *TNF-α*, and *MCP-1* were lower in galectin-12 knockdown macrophages activated with LPS (Figure 5d), OAPA (Figure 5e), or OxLDL (Figure 5f). ELISA assays confirmed that IL-6, TNF-α, and MCP-1 cytokines were downregulated in galectin-12 knockdown macrophages (Figure 5g). Taken together, our results suggested that galectin-12 plays important roles in human and mouse macrophage polarization.

## 3. Discussion

Leptin is a peptide hormone secreted by adipose tissues and its amount is generally positively associated with the amount of fat tissue. In a previous study, we found that Gal12^‒/‒^ mice exhibited lower amounts of adipose tissue compared to Gal12^+/+^ mice [8], which suggested lower leptin levels in Gal12^‒/‒^ mice. Elevated plasma leptin positively correlates with cardiovascular disease. Moreover, daily leptin administration to apolipoprotein E-deficient mice increased atherosclerotic lesions [15]. In this study, we found lower levels of plasma leptin in Gal12^‒/‒^ mice, suggesting that the ablation of galectin-12 may have a protective benefit against atherosclerosis.

OxLDL has been shown to induce inflammation through the activation of sequence-specific protein 1 (SP1) [16] and CCAAT/enhancer-binding protein (C/EBP) [17]. Both SP1 and C/EBP binding sites are present in the promoter region of galectin-12, suggesting that OxLDL induces galectin-12 expression via SP1 and C/EBP activation. Macrophages take up large amounts of OxLDL, which results in foam cell formation. Since the hallmark of early atherosclerotic lesions is the accumulation of macrophage-derived foam cells in the subendothelial space, we investigated foam cell formation in Gal12^+/+^ and Gal12^‒/‒^ mouse macrophages treated with OxLDL or AcLDL. We found significantly lower foam cell formation in Gal12^‒/‒^ compared to Gal12^+/+^ macrophages, indicating that galectin-12 ablation may indeed be beneficial in the pathogenesis of atherosclerosis.

Plasticity allows cells to shift morphology and behavior in response to an internal or external stimulant, which enhances differentiation to specific phenotypes [18]. The functions of galectin-12 indicate that this intracellular protein works as a cell plasticity modulator, enhancing cellular reprogramming to alter the biological function. The stimulation of macrophages by surrounding stimuli results in the polarization toward heterogeneous phenotypes and functions. M1 and M2 macrophages exhibit distinct functions and transcriptional profiles. It has been suggested that therapeutics could be delivered to promote macrophage polarization toward the M2 phenotype for the prevention or treatment of atherosclerosis [19]. Our results show that galectin-12 ablation in LDLRKO mice (double knockout) enhances M2 macrophage polarization. Macrophages engulf OxLDL or AcLDL through scavenger receptors, such as CD36. The ingested cholesterol ester is hydrolyzed to free cholesterol, which is then exported extracellularly to cholesterol acceptors by SRB1, ABCA1, or ABCG1 [20,21]. The significant downregulation of *CD36*, and the upregulation of *SRB1*, *ABCA1*, and *ABCG1* in DKO BMDM resulted in lower lipid droplet accumulation, thus abolishing foam cell formation.

MCP-1 plays an important role in inflammatory diseases, atherosclerosis, and cancer. OxLDL induces MCP-1 production and secretion by endothelial cells, smooth muscle cells, and macrophages [22]. Excessive levels of MCP-1 have been found in macrophage-rich atherosclerotic lesions [23,24]. Moreover, monocyte diapedesis to the subendothelial space and piling up of lipid-laden foam cells within the arterial wall are enhanced by MCP-1 [25]. MCP-1 has been identified in the initial phase of atherosclerotic lesion formation among the arterial specimens of patients, indicating that MCP-1 is involved in the early influx of monocytes into the arterial wall [26]. Upon infiltration, macrophages are activated by OxLDL and subsequently secrete inflammatory cytokines, such as TNF-α and IL-6, enhancing the progression of atherosclerosis. TNF-α and IL-6 have been shown to promote the expression of MCP-1 in macrophages [27,28]. The vicious cycle created between OxLDL macrophage activation, TNF-α and IL-6 macrophage secretion, and MCP-1-macrophage recruitment greatly boosts the development of atherosclerosis. In our study, we found significantly lower expression levels of MCP-1 in galectin-12 knockout mice and human macrophages, and compelling lower levels of macrophage infiltration in the atherosclerotic region.

Previously, Xue et al. found that galectin-12 is involved in the granulocytic differentiation of human promyelocytic leukemia cells [29]. Galectin-12 knockdown enhanced functional and morphological differentiation through all-trans retinoic acid, but inhibited lipid droplet accumulation. However, we could not reproduce these results using murine cell lines suggesting a different regulatory role of galectin-12 in humans and mice. Nevertheless, we investigated the role of galectin-12 in human macrophage polarization and the results were similar to those in mice macrophages.

In addition to modulating macrophage function, galectin-12 may also be involved in altering the function of endothelial cells or nitric oxide (NO) through inflecting insulin sensitivity [14]. Insulin resistance, often associated with adipokine dysregulation and hyperglycemia, promotes the secretion of vasoconstrictors and inflammatory molecules that result in enhancing endothelial dysfunction [30]. The malfunction of endothelial cells results in a flaw in the production or functioning of NO, an important molecule in endothelium-mediated vasodilation [30,31]. NO impedes the oxidation of LDL, which prevents foam cell formation [30,31]. Endothelial dysfunction is another important marker in the pathogenesis of atherosclerosis. The deletion of galectin-12 increases the activation of protein kinase A (PKA), which results in enhancing lipolysis in adipocytes [14]. Native glucagon-like peptide-1 (GLP-1) [32], GLP-1 agonist [32], eicosapentaenoic acid [33], and ApoA-I mimetic peptide D4-F [34] have been shown to promote M2 macrophage polarization by enhancing PKA activation. It would be interesting to determine whether PKA/cAMP is involved in M2 polarization in Gal12^‒/‒^ macrophages.

In conclusion, our results indicate that the ablation of galectin-12 leads to M2 macrophage polarization that consequently leads to reduced foam cell formation and proinflammatory cytokine production. These effects led to reduced atheroma formation in an atherosclerosis animal model. Therefore, we propose that inhibition of galectin-12 could be used as a therapeutic strategy to slow down the progression of atherosclerosis.

## 4. Materials and Methods

### 4.1. Animals and Animal Care

LDLRKO mice with a C57BL/6 background were purchased from Jackson Laboratory and were bred with galectin-12 knockout mice (C57BL/6 background) to obtain homozygous LDLR and galectin-12 double knockout (DKO) mice. Mice were maintained in normal chow diet until study initiation. The animal protocols were audited and approved by the China Medical University Animal Care and Use Committee (Approval No. CMUIACUC-2018-169-1). Mice were raised in ventilated cages (4 animals/cage) in a pathogen-free facility that provided a 12 h light/12 h dark cycle and fed a standard diet until 8 weeks of age. After 8 weeks of age, male LDLRKO and DKO mice were fed with either a low-cholesterol diet (LCD; 10% fat, 0% cholesterol) or high-cholesterol diet (HCD; 42% fat, 0.2% cholesterol) for 12 weeks. Overdose isoflurane anesthesia was used to sacrifice mice. Then, mice were perfused with phosphate buffered saline (PBS) and 10% neutral buffered formalin. The aortas and aortic roots were removed to determine atherosclerotic lesions.

### 4.2. Preparation of Bone Marrow-Derived Macrophages

Bone marrow cells (2 × 10^6^ cells/mL) isolated from Gal12^+/+^, Gal12^‒/‒^, LDLRKO and DKO mice were cultured in 10 cm petri dishes in RPMI medium (Merck KGaA, Darmstadt, Germany). Nonadherent cells were removed by replacing the culture medium on Days 3 and 5. The adherent cells at Day 7 were matured BMDM used for further experiments.

### 4.3. Cytokine Enzyme-Linked Immunosorbent Assay

Mice sera or THP-1 macrophage culture supernatants were collected and analyzed for cytokine content using commercially available enzyme-linked immunosorbent assay reagents for TNF-α, IL-6 and leptin, and were performed according to the manufacturer’s instructions (R&D system, Minneapolis, MN, USA).

### 4.4. Quantitative Polymerase Chain Reaction

Total RNA was obtained using an RNA isolation kit (Qiagen, Germantown, MD, USA) and reverse transcriptase (Invitrogen) was used to synthesize cDNA from 5 μg total RNA. The universal probe system (Roche Life Science) was used to perform real-time quantitative polymerase chain reaction (qPCR). The reference gene used for normalization of the RNA samples was glyceraldehyde 3-phosphate dehydrogenase. Primer pairs used to perform semi-quantitative PCRs were: 5′-CCTGCTCACGTGCTCTTCCTCG-3′ and 5′-TTGGAGCCCTTCTTAGCAGTGG-3′ for mouse galectin-12, and primers 5′-TGAAGGTCGGTGTGAACGGATTTGGC-3′ and 5′-CATGTAGGCCATGAGGTCCACCAC-3′ for mouse glyceraldehyde-3-phosphate dehydrogenase.

### 4.5. Immunofluorescence Staining

The THP-1 macrophage cells and BMDM were cultured on 16-well chamber slides. Cells were rinsed carefully with Tris-buffered saline (TBS), treated with 4% paraformaldehyde, and blocked with 1% bovine serum albumin and 0.1% Triton X-100 for 1 h. Polyclonal anti-galectin-12 antibody was applied to the cells and an Alexa Fluor 488 conjugated secondary antibody was used. The nuclei were revealed using a 4′,6-diamidino-2-phenylindole (DAPI) DNA stain. Fluorescence microscopy or confocal microscopy were used to obtain cell images. The anti-galectin-12 antibody was acquired through immunization of Gal12^‒/‒^ Balb/c mice with recombinant mouse galectin-12.

Frozen aortic root sections were labeled with Alexa Fluor 488 conjugated antibody against F4/80 (Abcam). The nuclei were revealed using a 4′,6-diamidino-2-phenylindole (DAPI) DNA stain. Fluorescence microscopy or confocal microscopy were used to obtain cell images.

### 4.6. Lipid Droplet Staining

Cells or tissues were washed 3 times with PBS and then fixed with formaldehyde. Fixed cells or tissues were stained with oil red O and hematoxylin and eosin. For fluorescent lipid droplet staining, AdipoRed^TM^ assay reagent (Lonza) was used, and the nuclei were stained with a DAPI DNA stain.

### 4.7. Generating Doxycycline-Inducible Galectin-12 Knockdown THP-1 Cells

Stable conditional galectin-12 knockdown THP-1 cells were generated using previously described methods [29,35]. Two different artificial microRNAs (siRNA 138 and siRNA 3991) were used to knockdown galectin-12. Artificial microRNA targeting the LacZ gene was used as control. The sequences used for each artificial microRNA are listed in Table 1. THP-1 cells were transduced with a retrovirus containing reverse tetracycline-regulated transactivator (rTTA), and then selected in 1 μg/mL blasticidin for 1 month. THP-1 cells expressing rTTA were then transduced with lentiviruses containing artificial microRNAs targeting the LacZ or galectin-12 genes, and selected in 1 μg/mL puromycin for 1 month. To induce the expression of the artificial microRNAs, THP-1 cells were treated with 1 μg/mL doxycycline for 3 days. To promote THP-1 cell differentiation into macrophages, the cells were treated with 200 nM phorbol myristate acetate (PMA) for 48 h.

### 4.8. Statistical Analysis

Differences in study groups were analyzed using ANOVA (*p* < 0.0001), and Dunnett’s multiple comparisons tests were used for paired comparisons between control and treated groups. *p* values less than 0.05 indicate statistical significance.

## Figures and Tables

**Table 1:** miRNA sequences used in this manuscript.

Name	Sequence
138top	5′-TGCTGACTCCTTGCAGCATGACCATCGTTTTGGCCACTGACTGACGATGGTCACTGCAAGGAGT-3′
138bottom	5′-CCTGACTCCTTGCAGTGACCATCGTCAGTCAGTGGCCAAAACGATGGTCATGCTGCAAGGAGTC-3′
3991top	5′-TGCTGAGAAAGTGCTGTCCATTCACAGTTTTGGCCACTGACTGACTGTGAATGCAGCACTTTCT-3′
3991bottom	5′-CCTGAGAAAGTGCTGCATTCACAGTCAGTCAGTGGCCAAAACTGTGAATGGACAGCACTTTCTC-3′

## Abbreviations

AcLDL	Acetylated LDL
ABCA1	ATP Binding Cassette Subfamily A Member 1
ABCG1	ATP Binding Cassette Subfamily G Member 1
CRD	carbohydrate recognition domain
BMDM	bone-marrow-derived macrophages
CD36	cluster of differentiation 36
cAMP	cyclic adenosine monophosphate
DKO	galectin-12 and LDL receptor double knockout
HCD	high-cholesterol diet
Il-6	interleukin-6
LPS	lipopolysaccharide
LCD	low-cholesterol diet
LDL	low-density lipoproteins
LDLRKO	LDL receptor knockout
MCP-1	monocyte chemoattractant protein-1
OxLDL	oxidized LDL
PMA	phorbol myristate acetate
PKA	protein kinase A
SRB1	scavenger receptor class B type 1
TNF-α	tumor necrosis factor α
DAPI	4′,6-diamidino-2-phenylindole
